# Supplementary material for: The interactome of intact mitochondria by cross-linking mass spectrometry provides evidence for coexisting respiratory supercomplexes
Source: Mol Cell Proteomics. 2017 Dec 8;17(2):216–32. doi: 10.1074/mcp.RA117.000470 (PMC5795388; doi:10.1074/mcp.RA117.000470)
Supplement: Supplemental Data [file supp_RA117.000470_133922_0_supp_23978_fzffwf.zip › spectra_annotation/mito_spectra_annotation/280-1-8-1-11-1.pdf]

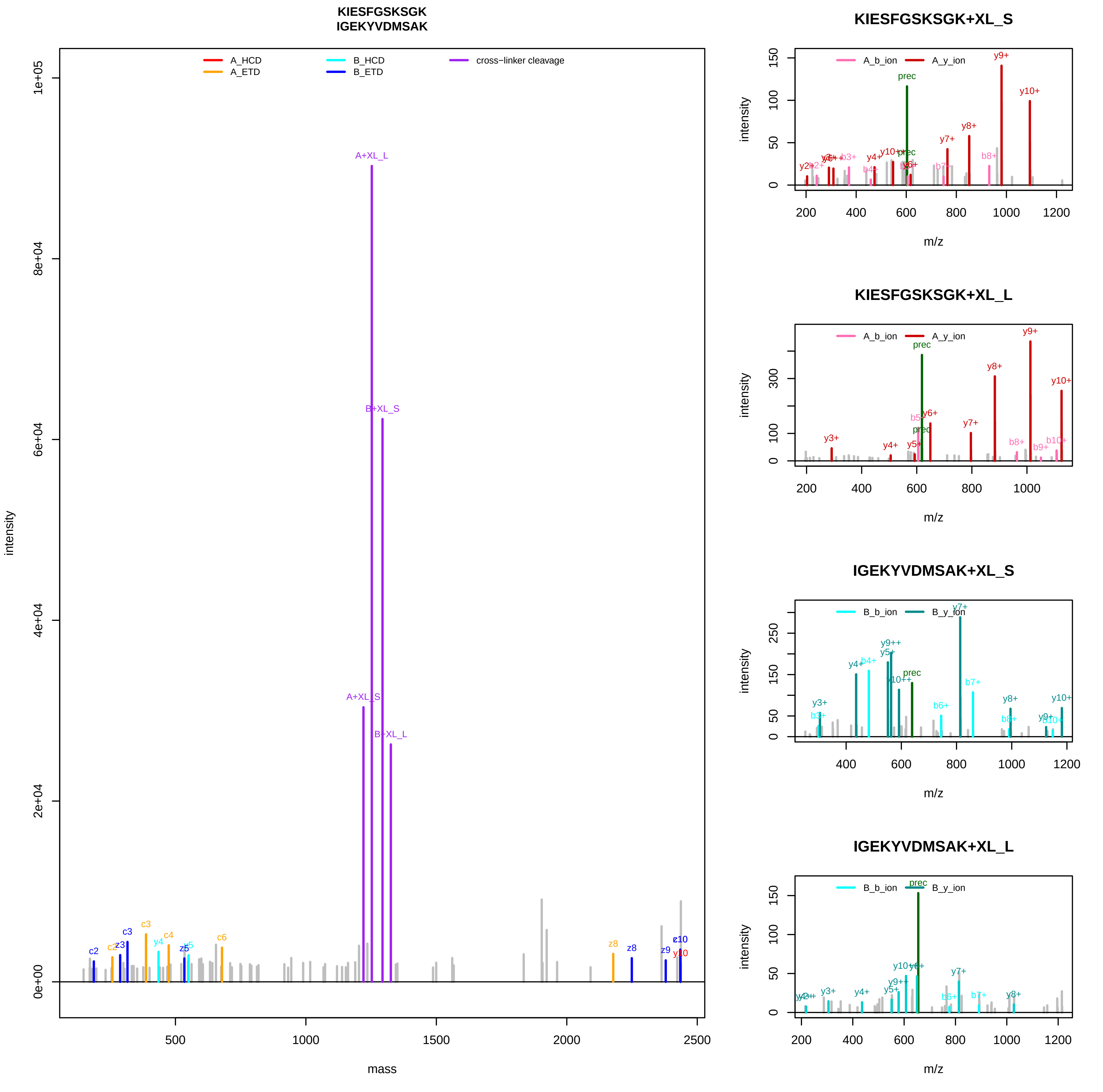
KIESFGSKSGK
IGEKYVDMSAK
A_HCD
A_ETD
B_HCD
B_ETD
cross−linker cleavage
A+XL_L
B+XL_S
A+XL_S
B+XL_L
c2
c3
z3
y4
c4
z5
c6
z8
z9
c10
y10
intensity
mass
KIESFGSKSGK+XL_S
A_b_ion
A_y_ion
prec
y9+
y10+
y8+
y7+
b8+
b7+
y4+
b4+
b3+
y2+
b2+
m/z
KIESFGSKSGK+XL_L
y3+
y5+
y6+
b5+
b9+
b10+
IGEKYVDMSAK+XL_S
B_b_ion
B_y_ion
y9++
y10++
b6+
m/z
IGEKYVDMSAK+XL_L
y2++
y9++
y10++
